# Axillary Lymph Node Dissection in Angiosarcomas of the Breast: An Asian Institutional Perspective

**DOI:** 10.1155/2020/4890803

**Published:** 2020-03-25

**Authors:** Sharanniyan Ragavan, Hui Jun Lim, Joey Wee-Shan Tan, Josephine Hendrikson, Jason Yongsheng Chan, Mohamad Farid, Claramae Shulyn Chia, Grace Hwei Ching Tan, Khee Chee Soo, Melissa Ching Ching Teo, Chin-Ann Johnny Ong

**Affiliations:** ^1^Department of Sarcoma, Peritoneal and Rare Tumours (SPRinT), Division of Surgery and Surgical Oncology, National Cancer Centre Singapore, Singapore 169610; ^2^School of Medical Sciences, Faculty of Biology, Medicine and Health, The University of Manchester, Manchester M13 9PL, UK; ^3^Division of Medical Oncology, National Cancer Centre Singapore, Singapore 169610; ^4^Duke-NUS Medical School, Singapore 169857

## Abstract

Angiosarcomas of the breast (ASB) are rare, making up to less than 8% of all angiosarcomas. The surgical management for this disease continues to vary throughout centres worldwide due to the current limited evidence. We aim to examine the necessity of axillary lymph node dissection in this pathology through a retrospective study of axillary metastasis and recurrence patterns in patients treated at our institution. A retrospective review of a prospectively-maintained database was performed. All adult patients with a histologically confirmed diagnosis of ASB seen at the National Cancer Centre Singapore between 2006 and 2019 were identified. Axillary lymph node status, treatment, survival, and recurrence data were collated. Thirteen patients were identified with a confirmed diagnosis of ASB, of which there were 11 primary and 2 secondary angiosarcoma cases. Eight patients had some form of axillary lymph node dissection and 5 did not. No positive nodes were found in any examined axillary nodes despite high median number of nodes harvested (13, range 8–24). 5/13 patients had disease progression, of whom none had locoregional recurrence to the axilla. ASB continues to be rare and recurrent and presents as a challenge to treat. Axillary lymph node involvement is most likely not present in a majority of patients. Prophylactic removal is unwarranted in patients presenting without lymph node involvement due to the lack of axillary metastasis.

## 1. Introduction

Angiosarcomas are malignancies arising from the endothelium of vascular channels [1]. They are a substantially rare subtype of sarcoma and make up to less than 1% of all sarcoma cases [1–3]. Although they have the potential to present anywhere in the body [2], they infrequently appear in the soft tissue of the breast, with angiosarcomas of the breast (ASB) estimated to form only about 8% [4] of all angiosarcomas. As a corollary, ASB forms only 1% [5, 6], if not lesser [3, 7–9], of all soft tissue breast malignancies. Hence, majority of the knowledge of this disease is summed from small case series, case reports, and aggregated reviews, leading to a lack of general consensus in the literature on the modality of treatment for ASB.

Multiple studies [1–3, 6, 7, 10–15] have reported a vastly varying range of treatments from wide excision to chest wall resection and radical mastectomy, while others [2, 8, 10, 12] suggest that simple mastectomy is the primary option. There is agreement, however, that nodal involvement is uncommon [1, 3, 5, 8, 10–13, 16] in this disease. This has been attributed to the predominantly hematogenous spread [5, 12, 17–19], which leaves the role of axillary lymph node dissection (ALND) in ASB previously unexplored. Several authors have reported data on ALND, but these studies [3, 10–12, 14, 16, 20] are limited in that there is a lack in one or more of the following: analysis of the role of ALND as a prognostic factor, data on angiosarcomas specific to the breast tissue, sufficient patient numbers, or complete ALND data for all patients.

Therefore, we conducted a retrospective review of our institution's database to examine the patterns of metastasis and recurrence in this disease, as well as note the rates of axillary nodal involvement, so as to better define the role of ALND in the management of ASB.

## 2. Materials and Methods

This study was approved by the SingHealth Centralized Institutional Review Board (CIRB 2018/3065/B). A retrospective review of our institution's prospectively-maintained database was performed. Thirteen patients (all female) who were diagnosed with ASB from 2006–2019 at National Cancer Centre Singapore were identified.

Patient demographics, clinicopathological, radiological, treatment and follow-up, and axillary lymph node information was extracted and tabulated with medians and ranges presented as appropriate. Tumour size was defined as the largest dimension of the lesion, while tumour grade and tumour differentiation were classified according to the French Federation of Cancer Centres Sarcoma Group guidelines. The diagnosis date of angiosarcoma was determined as the date of histological diagnosis following definitive surgery and survival duration as the time period from the date of surgery to last follow-up. Primary angiosarcoma was defined as a sarcoma arising *de novo* without prior radiation exposure while secondary angiosarcoma arose from a previously irradiated field. Axillary lymph node information collected included whether axillary dissection was performed and if so, what procedure was performed (ALND or sentinel lymph node biopsy (SLNB)), number of nodes harvested, and number of pathologically positive nodes for tumour metastasis.

## 3. Results and Discussion

### 3.1. Patient Characteristics

Characteristics of the 13 patients included in our study are summarized in Tables 1 and 2. The median follow-up time of all patients was 20.4 months (range: 1.0–133.6). The median age was 42 years (range: 26–70), and all patients were female. Majority of the patients were of Chinese descent (61.5%). There were 1 Indian and Malay patient (7.7%) each, with the remaining (23.1%) being classified as Others. All patients had Eastern Cooperative Oncology Group (ECOG) status of 0. 2 patients (patients 1 and 3) presented with secondary angiosarcoma as they had prior radiotherapy exposure for invasive ductal breast carcinoma. The median latency period amongst the 2 secondary angiosarcoma patients was 103.8 months, defined by the period between the date of radiotherapy initiation for primary breast cancer to the time of angiosarcoma diagnosis. The median age of patients with primary and secondary angiosarcoma were 40 (range: 26–57) and 56 (range: 42–70), respectively. The median tumour size was 5.0 cm (range: 1.3–23.0 cm) with 2 Grade 1 (15.4%), 5 Grade 2 (38.5%), and 4 Grade 3 (30.7%) tumours. Two tumours (15.4%) were of unknown grade. All patients were nonmetastatic (M0) at presentation to our institution.

### 3.2. Treatment

All patients underwent biopsy to confirm angiosarcoma diagnosis prior to curative surgery. Our centre has been performing routine prophylactic ALND for all suitable ASB patients. 7/13 patients (53.8%) underwent full ALND, while 1 patient (7.7%) had an SLNB (Table 1). This patient (patient 6) did not undergo ALND as she was discussed at the multidisciplinary tumour board meeting and was recommended for mastectomy and SLNB if determined to be lymph node-negative on the PET-CT scan. No positive lymph nodes were identified in any patient. 5/13 patients (38.5%) did not undergo ALND for ASB. Two patients had prior ALND for invasive ductal breast carcinoma. Another 2 patients underwent curative surgery at other institutions before presenting at our centre, while the last patient had reconstruction performed at the time of curative surgery and was not recommended for ALND. A total of 5 patients (38.5%) received adjuvant radiotherapy and 1 patient (7.7%) received adjuvant chemoradiotherapy. There were no reported 30-day complications in our patient cohort.

### 3.3. Recurrence

5/13 (38.5%) patients had recurrence (Table 1). Notably, none developed locoregional axillary recurrence. 2/8 primary ASB patients who underwent ALND developed distant recurrence. Patient 9 developed metastases to the contralateral breast, liver, and left ovary 6.9 months following surgery; however, she was lost to follow-up after. The other patient (patient 4) presented with bone metastasis to the right iliac bone 5.8 months following surgery and received palliative chemotherapy with a survival duration of 20.6 months. 2/3 primary ASB patients who did not undergo ALND-developed distant metastases. One had distant recurrence to liver, spleen, and peritoneum 6.8 months following surgery and passed away 1.2 months after (patient 11), while the other had distant recurrence to ovary, omentum, lung, and scalp 11.0 months following surgery, received palliative radiotherapy, and passed away 9.8 months after (patient 13).

Of the 2 secondary ASB patients, 1 patient (patient 1) had biopsy-proven local recurrence at the right medial breast and lateral chest wall occurring 4.4 months following curative mastectomy and ALND. Subsequently, she developed distant metastasis to the pleura 15.2 months following surgery and demised 17 days after.

## 4. Discussion

In our clinical audit where axillary node sampling and dissection were performed, we noted the absence of involved nodes. The median number of nodes harvested amongst these patients was also high (13, range: 8–24) reflecting that the lack of positive nodes was not due to poor lymph node harvest during axillary dissection. Moreover, no locoregional axillary recurrence was observed in our patient cohort. This suggests a low utility of axillary node dissection and axillary node sampling in angiosarcoma of the breast.

Prior to this review, our unit routinely performed prophylactic ALND for ASB patients; this decision was based upon previous analyses that reported high nodal involvement rates [16, 21, 22] in angiosarcoma. Therefore, almost all patients who were operated on at our centre had undergone prophylactic ALND for ASB.

The available literature on angiosarcoma of the breast is largely heterogenous, small, and inconclusive. Five-year overall survival rates ranged from 38–78% and 5-year recurrence-free survival between 38 and 56%, in cohort sizes of 16–69 [7, 11, 14]. Several reports have elucidated that ASB is an aggressive recurrent disease. Local/locoregional recurrence rates range between 25 and 62.5% [1, 3, 7, 11]. This contrasts with that of our results as only 1/5 patients (20%) recurred locally, although our sample size is too small to draw conclusive remarks. There are also conflicting results on differences in outcomes between primary and secondary angiosarcomas. Multiple studies [3, 7, 11] reported poorer outcomes in patients with secondary angiosarcomas, while Vorburger et al. [14] found no difference in long-term survival outcomes between angiosarcomas with and without prior radiation.

To the best of our knowledge, no analysis has been carried out on the necessity of routine ALND in the management of angiosarcomas of the breast. Majority of the previous studies on this disease have been focused exclusively [1–3, 8, 11, 14] on Western populations. Additionally, there are a limited number of analyses that have a sufficiently large cohort or an Asian perspective [22–24]. Thus, our series most significantly adds a single institutional Asian cohort to the literature and also explores the need for noncurative ALND by observing axillary metastasis and recurrence patterns.

Notably, our study is limited as a primarily descriptive study due to the continued rarity of this disease, our small cohort size, and its retrospective nature. As a further confounder, amongst the 5 patients who did not have ALND performed, 2/5 already had a prior ALND performed for their initial breast carcinoma. Nonetheless, this investigation is appreciably the first step in the creation of standard clinical guidelines for the management of angiosarcomas of the breast. We do implore the need for further studies for adjuvant molecular therapeutics on these patients.

## 5. Conclusions

In conclusion, we found no clinical or radiological evidence of axillary lymph node metastasis or locoregional recurrences in the axilla within our cohort. This was despite a high median number of lymph nodes harvested. Hence, we suggest that prophylactic ALND is unwarranted in patients with angiosarcoma of the breast who do not present with lymph node disease. Further studies for adjuvant molecular therapeutics should be considered.

## Figures and Tables

**Table 1:** Summary of patient characteristics.

Characteristics	Total (*n* = 13)	
	*n*	%
Female	13	100.0
Ethnicity		
Chinese	8	61.5
Indian	1	7.7
Malay	1	7.7
Others	3	23.1
Age at diagnosis (median (range))	42 (26–70)	
ECOG status 0	13	100.0
Type of angiosarcoma		
Primary	11	84.6
Secondary	2	15.4
Tumour grade		
1	1	7.7
2	4	30.8
3	1	7.7
Unknown	7	53.8
Tumour size (cm) (median (range))	5.0 (1.3–23.0)	
Type of curative surgery		
Nipple-sparing mastectomy	1	7.7
Radical mastectomy	4	30.8
Simple mastectomy	3	23.1
Wide excision	1	7.7
Completion mastectomy	4	30.8
Axillary lymph node dissection		
ALND	7	53.8
SLNB	1	7.7
No dissection	5	38.5
No. of lymph nodes retrieved (median (range)) ^*∗*^*n* = 6 (7 unknown/not applicable)	13 (8–24)	
Adjuvant radiotherapy	5	38.5
Adjuvant chemotherapy	1	7.7
Palliative radiotherapy	1	7.7
Palliative chemotherapy	1	7.7
Local recurrence	1	7.7
Distant recurrence	5	38.5

Abbreviations: ECOG, Easter Cooperative Oncology Group; ALND, axillary lymph node dissection; SLNB, sentinel lymph node biopsy.

**Table 2:** Summary of patient demographics, clinical presentation, treatment, and follow-up (*n* = 13).

Pt	Gender, age	Type of ASB	Tumour size (mm)	Tumour grade	Tumour differentiation	Prior radiation/RT latency period (months)	Treatment	ALND or SLNB (yes/no)	No. of recurrences	Time to recurrence (months)	Site of recurrence	Length of follow-up (months)
1	F, 70	Secondary	5.5	3	3	Yes/119.6	Completion mastectomy	No	2	4.4, 15.2	(1) Breast (right breast medial and lateral chest wall nodule) (2) Pleura	15.7
2	F, 32	Primary	8	2	2	No	Radical mastectomy	Yes	0	NA	NA	20.4
3	F, 42	Secondary	2	2	2	Yes/88.0	Completion mastectomy	No	0	NA	NA	61.1
4	F, 50	Primary	Unknown	3	Unknown	No	Radical mastectomy	Yes	1	5.8	Bone	20.6
5	F, 44	Primary	4.7	2	Unknown	No	Radical mastectomy	Yes	0	NA	NA	22.9
6	F, 57	Primary	1.3	1	1	No	Simple mastectomy	Yes (SLNB)	0	NA	NA	8.2
7	F, 44	Primary	5	1	Unknown	No	Wide excision	Yes	0	NA	NA	3.5
8	F, 40	Primary	Unknown	Unknown	Unknown	No	Completion mastectomy	Yes	0	NA	NA	133.6
9	F, 26	Primary	Unknown	Unknown	Unknown	No	Simple mastectomy	Yes	1	6.9	Right contralateral breast, liver, and ovary	7.0
10	F, 28	Primary	6.5	3	Unknown	No	Completion mastectomy	Yes	0	NA	NA	8.2
11	F, 43	Primary	23	3	2	No	Radical mastectomy	No	1	6.8	Liver, spleen, and peritoneum	8.0
12	F, 28	Primary	4.6	2	Unknown	No	Nipple-sparing mastectomy	No	0	NA	NA	1.0
13	F, 30	Primary	19	2	2	No	Simple mastectomy	No	1	11.0	Ovary, omentum, scalp, and lung	20.8

## Data Availability

The data used to support the findings of this study are included within the article.

## References

[1] Biswas T., Tang P., Muhs A., Ling M. (2009). Angiosarcoma of the breast. *American Journal of Clinical Oncology*.

[2] Abraham J. A., Hornicek F. J., Kaufman A. M. (2007). Treatment and outcome of 82 patients with angiosarcoma. *Annals of Surgical Oncology*.

[3] Fraga-Guedes C., Gobbi H., Mastropasqua M. G., Botteri E., Luini A., Viale G. (2012). Primary and secondary angiosarcomas of the breast: a single institution experience. *Breast Cancer Research and Treatment*.

[4] Abbott R., Palmieri C. (2008). Angiosarcoma of the breast following surgery and radiotherapy for breast cancer. *Nature Clinical Practice Oncology*.

[5] Arora T. K., Terracina K. P., Soong J., Idowu M. O., Takabe K. (2014). Primary and secondary angiosarcoma of the breast. *Gland Surgery*.

[6] Hui A., Henderson M., Speakman D., Skandarajah A. (2012). Angiosarcoma of the breast: a difficult surgical challenge. *The Breast*.

[7] Luini A., Gatti G., Diaz J. (2007). Angiosarcoma of the breast: the experience of the European institute of oncology and a review of the literature. *Breast Cancer Research and Treatment*.

[8] Monroe A. T., Feigenberg S. J., Mendenhall N. P. (2003). Angiosarcoma after breast-conserving therapy. *Cancer*.

[9] Wang X. Y., Jakowski J., Tawfik O. W., Thomas P. A., Fan F. (2009). Angiosarcoma of the breast: a clinicopathologic analysis of cases from the last 10 years. *Annals of Diagnostic Pathology*.

[10] Rosen P. P., Kimmel M., Ernsberger D. (1988). Mammary angiosarcoma: the prognostic significance of tumor differentiation. *Cancer*.

[11] Sher T., Hennessy B. T., Valero V. (2007). Primary angiosarcomas of the breast. *Cancer*.

[12] Steingaszner L. C., Enzinger F. M., Taylor H. B. (1965). Hemangiosarcoma of the breast. *Cancer*.

[13] Bousquet G., Confavreux C., Magné N. (2007). Outcome and prognostic factors in breast sarcoma: a multicenter study from the rare cancer network. *Radiotherapy and Oncology*.

[14] Vorburger S. A., Xing Y., Hunt K. K. (2005). Angiosarcoma of the breast. *Cancer*.

[15] Jallali N., James S., Searle A., Ghattaura A., Hayes A., Harris P. (2012). Surgical management of radiation-induced angiosarcoma after breast conservation therapy. *The American Journal of Surgery*.

[16] Fong Y., Coit D. G., Woodruff J. M., Brennan M. F. (1993). Lymph node metastasis from soft tissue sarcoma in adults analysis of data from a prospective database of 1772 sarcoma patients. *Annals of Surgery*.

[17] Gaballah A. H., Jensen C. T., Palmquist S. (2017). Angiosarcoma: clinical and imaging features from head to toe. *The British Journal of Radiology*.

[18] Tang T., Li H. (2018). Repeated resection-associated breast angiosarcoma. *Medicine*.

[19] Lim R. F., Goei R. (2007). Angiosarcoma of the breast. *RadioGraphics*.

[20] Antman K. H., Corson J., Greenberger J., Wilson R. (1982). Multimodality therapy in the management of angiosarcoma of the breast. *Cancer*.

[21] Bae S. Y., Choi M.-Y., Cho D. H., Lee J. E., Nam S. J., Yang J.-H. (2011). Large clinical experience of primary angiosarcoma of the breast in a single Korean medical institute. *World Journal of Surgery*.

[22] Shet T., Malaviya A., Nadkarni M. (2006). Primary angiosarcoma of the breast: observations in Asian Indian women. *Journal of Surgical Oncology*.

[23] Lokanatha D., Anand A., Lakshmaiah K. C. (2018). Primary breast angiosarcoma - a single institution experience from a tertiary cancer center in South India. *Breast Disease*.

[24] Kunkiel M., Maczkiewicz M., Jagiello-Gruszfeld A., Nowecki Z. (2018). Primary angiosarcoma of the breast- series of 11 consecutive cases—a single-centre experience. *Current Oncology*.

